# Human brain organoid code of conduct

**DOI:** 10.3389/fmmed.2023.1143298

**Published:** 2023-03-23

**Authors:** Meagan Hoppe, Ahmed Habib, Riya Desai, Lincoln Edwards, Chowdari Kodavali, Natalie Sandel Sherry Psy, Pascal O. Zinn

**Affiliations:** ^1^ UPMC Hillman Cancer Center, University of Pittsburgh, Pittsburgh, PA, United States; ^2^ Department of Neurosurgery, University of Pittsburgh, Pittsburgh, PA, United States; ^3^ Department of Neurology, University of Pittsburgh Medical Center, Pittsburgh, PA, United States; ^4^ Department of Hematology University of Pittsburgh Medical Center, Pittsburgh, PA, United States; ^5^ Department of Neurological Surgery, University of Pittsburgh, Pittsburgh, PA, United States

**Keywords:** organoids, ethics, consciousness, cancer biology, culture

## Abstract

Human brain organoids are models derived from human embryonic or induced pluripotent stem cells that mimic basic cerebral microanatomy and demonstrate simple functional neuronal networks. Brain organoids have been a rapidly expanding avenue for biomedical research in general and specifically: neural development, regeneration, and central nervous system pathophysiology. However, technology replicating functional aspects of the human brain, including electrically active neural networks, requires a responsible code of conduct. In this review, we focus the discussion on intrinsic and extrinsic ethical factors associated with organoids: intrinsic considerations arise with the growing complexity of human brain organoids, including human-animal chimerism, consciousness development, and questions of where these human-like beings fall in a moral hierarchy. Extrinsic considerations explore ethics on obtainment, manufacturing, and production of sophisticated human products. In summary, a thoughtful code of conduct using human brain organoids towards the advancement of science and medicine is crucial. This article shall facilitate a structured thought process approaching the moral landscape of organoid technology.

## Introduction

The human central nervous system (CNS) is highly complex and coordinated, responsible for perceiving, integrating, and processing information related to the entire body. (Mansour et al., 2021). Development of the CNS is an intricate process that involves communication between numerous neural and non-neural cell types and precisely timed factors that lead to proper migration, spatial differentiation, and functional organization. (Stiles and Jernigan, 2010; Mansour et al., 2021). Traditional 2D monolayer cultures and animal models do not fully recapitulate these complex multicellular interactions and thus limit efforts to further investigate human development and disease *in vitro*. (Mak et al., 2014; Mansour et al., 2021). Moreover, preclinical findings in these models often are not successful in achieving clinical significance. (Mak et al., 2014). However, advancements in stem cell technology have led to the development of brain organoids, which are self-organizing 3D models derived from pluripotent stem cells (PSCs). (Sakaguchi et al., 2015; Jo et al., 2016; Xiang et al., 2019; Mansour et al., 2021). These 3D models better resemble the architecture of specific brain regions and therefore may provide an improved *in vitro* framework for a variety of research applications. (Sakaguchi et al., 2015; Jo et al., 2016; Xiang et al., 2019; Mansour et al., 2021).

Numerous studies have utilized developmental signaling factors such as bone morphogenetic proteins (BMPs), Wnt, and sonic hedgehog in specific gradients to pattern PSCs into distinct dorsal, telencephalic (Cederquist et al., 2019; Ben-Reuven and Reiner, 2020), or mesencephalic fates, including the hippocampus (Sakaguchi et al., 2015) and midbrain (Jo et al., 2016), with regional functionality. Moreover, the fusion of these region-specific organoids allows for even more complexity; Xiang et al. have fused thalamic- and cortical-like organoids and demonstrated the development of reciprocal projections that mimic connections found *in vivo*. (Xiang et al., 2019). Furthermore, the long-term culture of organoids can model brain development; 10-month-old organoids have electroencephalogram signatures resembling preterm babies. (Trujillo et al., 2019). As these organoids increase in complexity, they provide opportunities for research in human brain aging, degeneration, and disease. Organoid models have already been applied to Parkinson’s disease, (Kim et al., 2019), amyotrophic lateral sclerosis, (Osaki et al., 2018), Alzheimer’s disease, (Choi et al., 2014; Gonzalez et al., 2018), traumatic brain injury, and brain tumors (Bian et al., 2018; Ogawa et al., 2018; da Silva et al., 2018). These models offer avenues for therapeutic advancements, such as screening brain tumor organoids for anti-tumor drugs. (Bian et al., 2018; Linkous et al., 2019). Commercial and public interest continues to increase as researchers demonstrate organoids as useful tools for drug screening and therapeutic targeting. (Chakradhar, 2016; Boers and Bredenoord, 2018; Choudhury et al., 2020; Hyun et al., 2020; Lensink et al., 2021).

As advancement continues and commercial interests rise, it becomes critical to consider the ethical issues surrounding the obtainment, modification, and manufacturing of human biomaterials. Moreover, as organoids become increasingly relevant in the study of human diseases, problems arise regarding patient privacy and benefit-sharing of pertinent clinical results to patient donors. (Aalto-Setälä et al., 2009; Boers et al., 2015; Boers and Bredenoord, 2018; Lensink et al., 2021). An examination of the strengths and limitations of current ethical guidelines for human biomaterial studies will aid researchers, ethicists, and industry in navigating the changing moral landscape of organoid research.

Brain organoids present novel concerns that have captured the interest of several scientists and ethicists. (Lavazza and Massimini, 2018; Shepherd, 2018; Chen et al., 2019; Lavazza, 2021). A recent panel report from the National Academies of Science, Engineering, and Medicine found that neural organoids are limited in complexity, maturity, and size and argued “it is extremely unlikely that in the foreseeable future they would possess capacities that, given current understanding, would be recognized as awareness, consciousness, emotion, or the experience of pain.” (National Academies of Sciences et al., 2021). However, as organoids begin to progress in complexity, some argue the distant future possibility of primitive consciousness development or self-awareness in organoids or animals implanted with organoids (Lavazza and Massimini, 2018; Shepherd, 2018; Lavazza, 2021). These concerns elicit new ethical problems of moral status, and question what obligations researchers have in protecting the moral rights of organoids. (Lavazza and Massimini, 2018; Shepherd, 2018; Lavazza, 2021).

Currently, the literature lacks a comprehensive review containing key ethical concerns for brain organoid research and proposed solutions. This review seeks to bridge this gap by collating these concerns into intrinsic and extrinsic considerations. Intrinsic considerations describe theoretical concerns that may arise with further organoid advancement, including the ethical creation and experimentation of human-animal chimeras and the development of conscious cerebral organoids with the potential for pain and suffering. Extrinsic considerations include those involved in the ethical collection of human materials and the downstream production and commercialization of their products. This discussion will provide a framework for a thoughtful code of conduct for ethical organoid research.

## Intrinsic ethical considerations

### Brain organoid animal transplantation—Human-animal chimerism

A major limitation in brain organoid development is the lack of vascularization. (Mansour et al., 2018; Pham et al., 2018). While recent efforts have demonstrated endothelial cells forming vascular networks in brain organoids, they have limited functionality. (Pham et al., 2018; Cakir et al., 2019). Thus, brain organoids are limited to a size of a few millimeters due to the high metabolic demand of neurons and their progenitors. (Mansour et al., 2018; Pham et al., 2018; Cakir et al., 2019). One solution is the transplantation of organoids into an animal host, which then can perfuse nutrients and enable further growth and maturation of the organoids. (Mansour et al., 2018). However, this cross-species transplantation has many ethical considerations, especially with brain organoids. The organoids have the potential to integrate into the CNS of the animal which could allow them to cognitively develop beyond what is possible for their species. (Chen et al., 2019). This creation of a “humanized” animal elicits questions about the moral status of these chimeras relative to their original species and what limitations we should place on their creation and experimentation.

Chimeras. The term “chimera” is broadly defined as consisting of any animal transplanted with human cells. (Chen et al., 2019; Koplin and Massie, 2020). Thus, organoid transplantation is a sub-category of chimerism. Neural transplantation has been performed as early as 1890 (Thompson, 1890), with numerous methodologic advancements and research interest expanding in the 1970s onward. (Björklund and Stenevi, 1985). Currently, guidelines for human chimera research are vague, and ethical oversight for organoid transplantation research is lacking. The National Academy of Sciences advises oversight committees for any study that introduces human PSCs (hPSCs) into non-human primates (NHP) or embryonic animals that have the potential to create an adult chimera. (National Research Council (US) and Institute of Medicine (US) Human Embryonic Stem Cell Research Advisory Committee, 2010). The International Society of Stem Cell Research similarly advises oversight to these studies with a specific focus on the potential for integration of human cells into animal hosts. (Lovell-Badge et al., 2021). Greene et al. suggested that these oversight committees specifically consider six factors, including the ratio of grafted cells to the host, integration, and development, host species, brain size, location of graft, and pathology. (Greene et al., 2005). Overall, there is limited governance in place for specifically brain organoid transplantation, but more nuanced approaches like those suggested by Greene et al. would allow for study-specific consideration of relevant ethical issues. For example, transplantation of an entire brain organoid into a mouse would create a brain with, at best, 0.5% the number of cells in an adult human brain, (Chen et al., 2019), thus it may not be possible for mice to ever reach the cognitive complexity of humans. Additionally, while recent work has demonstrated functional integration with existing structures including the visual cortex, (Wilson et al., 2022), currently, transplanted organoids into rodent hosts shows low integration, making this possibility even less likely. (Mansour et al., 2018; Revah et al., 2022). However, in NHPs with larger brains and a greater degree of cerebral complexity, it raises the concern of the “humanization” of NHPs (Chen et al., 2019). Therefore, more discussions should occur surrounding the ways in which brain organoid transplantation studies should be monitored and evaluated to ethically conduct experimentation without hindering scientific advancement.

Animal Enhancement. Chen et al. argue that in discussions on chimerism, it is less important to debate the similarity of chimeras to humans, and instead, emphasize a shift to discussions on what brain enhancements are possible and which have moral implications. (Chen et al., 2019). Already, researchers are demonstrating functional enhancement; for example, one study demonstrated that transplantation of hPSC-derived neurons into a stroke cavity showed functional response to sensory stimulation, (Tornero et al., 2017), and another showed that cerebral organoid transplantation in rats that had suffered traumatic brain injury improved their neural motor function and reduced brain damage. (Wang et al., 2020). Thus, it is important to frame which human-like enhancements (e.g., experience of pain, suffering, self-awareness) may render further experimentation morally unacceptable. Chen et al. proposed a pyramid of enhancements, with basic neurological functions such as movement or sensation at the bottom, cognitive functions higher, and self-awareness at the peak. (Chen et al., 2019). When monitoring the development of any functional improvements in chimeras, the moral value of the enhancement and its hierarchical position in the pyramid may require differential scrutiny. Once it is determined which enhancements are possible and moral status has been assigned, it is critical to develop objective measures to test the emergence of such enhancements in chimeras. Currently, some studies have investigated the existence of the antecedents of consciousness in animals, including metacognition, which is the awareness of and ability to control one’s own cognition, (Smith, 2010), and empathy, which exists on a continuum of complexity based on the species’ ability to perform higher-order cognitive processes. (de Waal and Preston, 2017). In future ethical oversight, it may become useful to incorporate these tests to monitor the emergence of heightened cognitive ability.

Self-Awareness. The peak of “humanization” is thought to be self-awareness, which is difficult to test given our lack of understanding of the neural circuitry and mechanisms that underlie the concept. (Shepherd, 2018; Chen et al., 2019; Lavazza, 2021). Self-awareness can be defined as diffuse advanced processing that provides information necessary for meta-cognition, allowing an organism to consciously control its behavior and recognize ownership of its experiences of the environment. (Lou et al., 2017). The current standard for measuring self-awareness among animals is the mirror test, in which the ability of an animal (or human) to recognize its own reflection is assessed; (Chen et al., 2019); only children around 2 years (Amsterdam, 1972), chimpanzees (Gallup, 1970), bottlenose dolphins (Reiss and Marino, 2001), Asian elephants (Plotnik et al., 2006), and magpies (Prior et al., 2008) have successfully passed the test to date. Although the mirror test is an indirect measure of self-awareness that potentially offers an observable indication of human-like mental function in chimera enhancement, this rudimentary test does not fully capture the concept of sentience as experienced by humans. Research efforts to better characterize and comprehensively test self-awareness should be emphasized.

Moral Status. It is critical to consider the welfare of chimeras after enhancement. If these animals develop more sophisticated cognitive behavior, it may be unethical to keep them in unstimulating environments. (Chen et al., 2019). Within the realm of neurorights proposed by Lenca and Yuste, mental integrity is a foundational principle. (Ienca, 2021). A key aspect of mental integrity is the prevention of psychologic harm, and thus as conversation for organoid ethical oversight evolves it may become important to examine how the right to mental integrity applies to chimeras. Similarly, if the human cells within the animal’s brain develop their own “detached consciousness,” it may become important to consider their wellbeing in addition to that of the animal. The sacrifice of animals for tissue studies may be morally unacceptable as they develop to become more “human-like,” and stricter guidelines might be needed for minimizing the pain and suffering of enhanced animal subjects. If these chimeras develop self-awareness, and experimentation is considered unethical, perhaps they could be retired from laboratory settings to animal sanctuaries. Currently, regulations on animal research do not consider self-awareness, yet there are advocates for greater restrictions. (Chen et al., 2019).

Resurrection. Beyond moral obligations to the living, it is now becoming critical to examine the moral rights of deceased animals and how organoid transplantation might challenge them. For instance, a recent study applied a pulsatile perfusion system that restored and maintained the neurophysiology of a pig brain with prior circulatory arrest for 4 h. (Vrselja et al., 2019). While not involving brain organoids, it illustrates the possibility of the resurrection of brain function and tissue. As organoid technology further develops, it may be possible to graft new neural tissue into areas of pathology or damage and restore brain function. In this new realm of innovation, it is critical to examine the ethical considerations of this experimentation and determine our moral obligations to both living and dead animals. (Farahany et al., 2019; Koplin and Massie, 2020).

### Consciousness development

Cerebral organoid research has rapidly advanced in the past decade, with organoids gaining more sophistication and complexity. As organoid technology improves and these systems begin to model the development and maturation of a human brain *in vivo* more closely*,* the potential of these entities to develop “consciousness” emerges as a key ethical issue. This is an ethical dilemma unique to brain organoids, as the brain is considered by most to be the root of human consciousness; for example, in clinical settings ‘brain death’ is considered an endpoint regardless of the health of the rest of the organ systems. Thus, in this section, we will explore theories of consciousness and their applicability to brain organoids, proposed methods to measure the development of basic ‘consciousness,’ and discuss how consciousness is linked to moral status and rights of organoids as entities.

Theories of Consciousness. The precise definition of “consciousness” is a contested topic with different schools of thought among researchers, philosophers, theologists, cognitive neuroscientists, *etc.* In a clinical context, the term “consciousness” can be used to refer to the level of arousal as well as reference the content of human experience. As such, on a basic scientific level, consciousness could be described as the activity of a neural network based on the stimulation of a specific region (e.g., reticular formation), while on the other hand, consciousness can be conceptualized as a complex, global integration of cortical and subcortical circuitry to produce human sentience. (Berlucchi and Marzi, 2019; Hyun et al., 2020). A popular theory of consciousness in the context of organoid research is the Integrated Information Theory (IIT), which details which processes are required to support consciousness. (Tononi et al., 2016; Lavazza and Massimini, 2018). The IIT postulates that consciousness is dependent on both differentiation and integration within neural circuits in the brain. (Tononi et al., 2016; Lavazza and Massimini, 2018). Specifically, it states that conscious experience is a) informative, meaning that each experience is unique and b) integrated, such that conscious experiences cannot be split into more fundamental parts. (Tononi et al., 2016; Lavazza and Massimini, 2018). It details that consciousness does not require the presence of sensory stimuli from an external environment, intact executive functioning, nor measurable motor output as long as the entity can integrate information. (Tononi et al., 2016; Lavazza and Massimini, 2018). Further, the IIT defines a physical substrate of consciousness, which is the physical manifestation of experience. (Tononi et al., 2016; Lavazza and Massimini, 2018). In other words, an experience occurs, and this changes some observable state within the entity that defines the conscious awareness and integration of that experience. This definition provides a framework for measuring consciousness in a physical manner and is preferred in discussions of organoid ethics over more philosophical definitions of consciousness that are too obscure to be measured.

Some researchers and ethicists suggest shifting the focus from defining consciousness towards understanding the biopsychological architecture of consciousness. (Shepherd, 2018). These neural correlates of consciousness (NCCs) seek to explain the physical brain structures and circuits that are responsible for consciousness *in vivo.* (Shepherd, 2018; Lavazza, 2021). From this perspective, researchers could monitor the development of specific circuitry or patterns in organoids that would indicate consciousness without having to precisely define what consciousness *is.*

Assessing Consciousness. If we accept that it is possible for brain organoids to gain consciousness, then the question of how to assess consciousness in an entity that cannot communicate emerges. The Perturbational Complexity Index (PCI) is based on the IIT and utilizes indices of neuronal functioning rather than direct motor or verbal response to determine conscious awareness. (Lavazza and Massimini, 2018). PCI is calculated by first applying a transcranial magnetic stimulation to a patient (or organoid) then measuring the magnitude and complexity of the electrical response using EEG. (Lavazza and Massimini, 2018). A low PCI would indicate either a lack of integration or differentiation and thus, by the IIT, a lack of consciousness. (Lavazza and Massimini, 2018). If the stimulation evokes a response that is spatially restricted, then it would lack ‘integration’ and indicate few interactions between neural circuits or regions. (Lavazza and Massimini, 2018). If the evoked response has a large magnitude but is ‘stereotypical’ such that it is the same across different regions then it lacks ‘differentiation,’ indicating the absence of the complexities of true *in vivo* neural communications. (Lavazza and Massimini, 2018). Therefore, PCI is high when a stimulation evokes a large response across regions that each reacts in a specialized way, which would suggest consciousness as defined by IIT. (Lavazza and Massimini, 2018). The PCI was validated in patients in NREM sleep or under anesthesia, finding that PCI was higher in those who reported conscious experience or awakening and lower in those who did not report conscious awareness. (Casarotto et al., 2016). Therefore, the adaptation of the PCI for stimulation and recording in organoids may be useful in the future for assessing consciousness.

**FIGURE 1:**
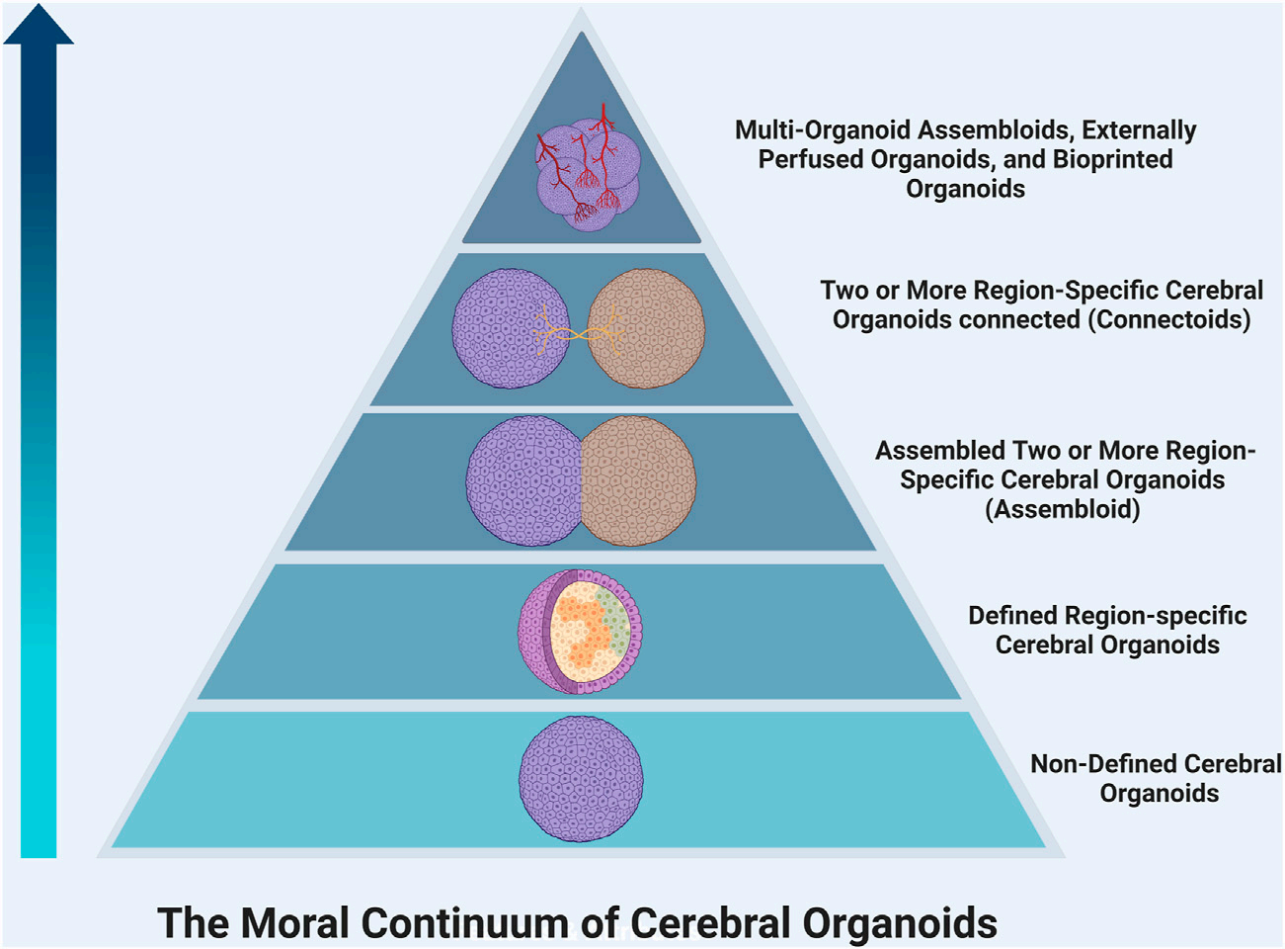
The continuum of moral significance in organoid research. Organoids vary widely in their complexity, so it becomes important to consider if, and where, different organoids fall on this continuum of moral significance. For example, an organoid using a protocol, which generates a cerebral organoid with varying populations of cells from different brain regions, would likely be lower on the hierarchy than a region-specific organoid, which contains more mature organization. Moving up the pyramid, co-culturing region-specific organoids creates reciprocal connections that begin to form primitive circuits. Connectoids offer even further maturation of function. The addition of more region-specific organoids, non-neural cell types like endothelial cells or microglia, external perfusion systems, and bioengineering will allow organoids to grow in size, cell number, and synaptic density. This will continue to increase the complexity and thus the moral significance of such organoids.

Moral Significance of Consciousness The development of consciousness in organoids would bring more ethical questions; most importantly, what is the moral significance of consciousness? It is important to begin discussions on the moral status of organoids to determine what rights they possess and what obligations we as researchers have to protect them. These discussions could be guided by first determining the value that we place on different conscious experiences. Many consider self-awareness to be the most complex form of consciousness and what distinguishes humans from other animals. (Shepherd, 2018; Hyun et al., 2020; Lavazza, 2021). Currently, there is debate about the ability of organoids to develop self-awareness; some believe it is possible, while others suggest that the lack of stimulatory inputs, social environment, and language acquisition would prohibit any organoid from truly realizing this potential. (Hyun et al., 2020). As such, organoids may be placed below living humans on a ‘moral hierarchy,’ but this does not necessarily exclude them from having rights that should be protected. Additionally, organoids vary widely in their complexity, so it becomes important to consider if, and where, different organoids fall on this continuum of moral significance. For example, initial neural organoid models contained several neuronal cell populations from different anatomic regions lacking sophisticated organization or mature circuits. (Lancaster and Knoblich, 2014). However, later studies have created primitive spatially organized cerebral organoids (Ben-Reuven and Reiner, 2020), assembloids (Xiang et al., 2017) -- in which two or more brain region-specific organoids are co-cultured–and connectoids (Cullen et al., 2019; Kirihara et al., 2019; Adewole et al., 2021), in which region-specific brain organoids are cultured separately but allowed to form reciprocal axonal connections and shown to develop more complex neural activity. As such, these more complex organoids have greater potential to develop primitive awareness and may be awarded greater moral significance (Figure 1). Also, these organoids may have increased potential to create chimeras with enhanced abilities when transplanted, and therefore should be approached more cautiously. We believe reframing discussion of consciousness and moral status in organoids from an all-or-none approach towards a more nuanced perspective with a moral continuum is beneficial to researchers and regulatory bodies. It may provide a framework for determining which studies require more ethical oversight in the future.

Taking a consequentialist perspective suggests weighing consequences against each other; in the case of organoid research with the potential to save the lives of many patients, a consequentialist may recommend that it is morally just to sacrifice the smaller, simpler life-form. (Lavazza and Massimini, 2018). However, it is also important to consider the potential for organoids to develop pain and researchers’ ethical obligations to minimize the suffering of living beings for scientific purposes. (Lavazza and Massimini, 2018; Lavazza, 2021). Similar to guidelines that protect research animals from undue pain or suffering, it may become important to set experimental rules that protect organoids. This initiative would be challenging due to the inability to communicate with organoids or visualize physical responses to pain.

## Extrinsic ethical considerations

### Ethical obtainment of human biomaterials

Organoid creation begins with the acquisition of human biomaterials to develop stem cell lines. (Boers and Bredenoord, 2018; Hyun et al., 2020). The ethical collection of biomaterials for research has traditionally involved two key ideas–obtaining donor consent or de-identification of samples–yet the evolution of technology renders these concepts inadequate. (Mostert et al., 2016; Boers and Bredenoord, 2018; Hyun et al., 2020). The transformation of simple human tissue into sophisticated organoid technology gives organoids not only biological and clinical potential but also moral and commercial value. In this new realm of innovation, it is important to examine the limitations of current research ethics and begin a discussion of better models to protect and balance the interests of donors, researchers, private industry, and society.

Consent. While it is widely accepted that consent is a necessary component of human tissue collection, debate ensues on which consent method is best. Consent methods vary mostly in the extent of detail provided on the future use of collected samples, ranging from ‘specific consent,’ that describes each project the tissue will be used for, to ‘blanket consent,’ in which donors provide samples for any future use without limitations. (Lowenthal et al., 2012; Boers and Bredenoord, 2018). The hurdle in this variation is that often donors do not give explicit consent for the derivation of stem cell lines or brain organoids from their samples, and as this research could be ethically controversial, donors may object. (Hyun et al., 2020). This is especially prevalent in tissue banks; most do not include the potential for the derivation of brain organoids in the consenting process and subsequent de-identification of samples makes it difficult to re-consent in the future. (Hyun et al., 2020). To address these concerns, some have proposed the concept of consent for governance, which would be a new paradigm that shifts the ethical emphasis from the initial consenting process to ongoing obligations for governance. (Boers and Bredenoord, 2018). Initial consent would consist of informing participants of any known future uses along with information on the governance structure that will be in place. (Boers et al., 2015; Boers and Bredenoord, 2018). This proposed governance structure includes transparency on management of data and tissue, identification of potential commercial uses, details on how clinical benefit will be translated to the donor, and a plan for longitudinal ethical oversight with designated committees. (Boers et al., 2015; Boers and Bredenoord, 2018). More research into donor preferences is needed to guide this debate on fair governance infrastructure. (Boers and Bredenoord, 2018; Hyun et al., 2020).

Protection of Privacy. De-identification of data and donor confidentiality have also been critical aspects of ethical research, yet some argue that anonymization is neither possible nor preferable. (Mostert et al., 2016; Boers and Bredenoord, 2018). With high-throughput genome sequencing, patient samples can become identifiable even after they have been anonymized. (Aalto-Setälä et al., 2009; Mostert et al., 2016; Boers and Bredenoord, 2018). This issue is emphasized in the perspective of neurorights, of which the right to privacy, particularly of neural data, is a conceptual foundation. (Ienca, 2021). Also, anonymization prevents not only the ability to re-consent donors for future use but also hinders patient and donor ability to share benefits from organoids generated from their tissues. (Boers et al., 2016; Boers and Bredenoord, 2018). This includes sharing findings with potential clinical benefits for the patient. (Boers et al., 2016; Boers and Bredenoord, 2018). De-identification also inhibits researchers from pairing the results from organoid research to clinical information or from developing precision medicine approaches to research. (Boers et al., 2016; Boers and Bredenoord, 2018; Choudhury et al., 2020). New proposed guidelines suggest a shift in focus from complete de-identification to development of measures like the ‘professional need to know’ basis of confidentiality in clinical practice. (Boers and Bredenoord, 2018). Before samples are obtained, researchers must ensure that privacy infrastructure is in place to protect patient data and that only relevant and necessary personal data are included. (Mostert et al., 2016; Boers and Bredenoord, 2018).

### Commercialization of organoids

For many years, animal models were the gold standard for *in vivo* experimentation; however, a major issue is the translatability to humans. (Boers et al., 2016; Choudhury et al., 2020; Hyun et al., 2020). Often, promising drugs or other therapies in animal studies ultimately fail in human clinical trials. (Boers et al., 2016; Choudhury et al., 2020; Hyun et al., 2020). Many believe organoids may fill this gap, and consequently, the interest in business and commercialization has surged. (Boers et al., 2016; Chakradhar, 2016; Choudhury et al., 2020; Hyun et al., 2020; Lensink et al., 2021). This elicits ethical issues of property rights, biobanking and manufacturing of human products, and benefit sharing in the commercialization and translation of organoid research and discovery.

Property Rights. The foundation of commercialization in research is the concept of intellectual property rights. (Roberts et al., 2014). Due to efforts to avoid commercialization of the human body, typically patients and donors lack property rights of their tissue. (Boers et al., 2016; Boers et al., 2019). However, with advancements in genomic techniques, researchers can now obtain and manipulate cell lines or tissues to produce a more complex product with the potential for profit that donors lose all rights to. (Boers et al., 2016). The intersection of human biomaterial research and intellectual property raises several ethical considerations. First, to what extent are human biomaterials patentable? The US Supreme Court decision in the case of *Association for Molecular Pathology et al. v. Myriad Genetics, Inc., et al.* ruled that the creation of new products from human biomaterials, such as the genetic manipulation of cell lines, alteration of genes, or creation of entities that are not naturally occurring are patent-eligible. (Roberts et al., 2014). Therefore, the creation of induced PSC lines and generation of organoids are legally qualified for patent claims. However, the field has already started rapid privatization in the early, preclinical stages of research. (Roberts et al., 2014). This has tremendous implications for increased costs that both could impact progress and restrict patients from accessing monetary or clinical benefits of organoid research. (Roberts et al., 2014).

Biobanking and Benefit Sharing. Organoid technology has captured the interest of numerous companies dedicated to different aspects of translation and the use of organoids. (Choudhury et al., 2020). For example, Organome (Chakradhar, 2016) and Hubrecht Organoid Technology (HUB) aim to create organoid biobanks, SUN Biosciences and System one Biosciences seek to improve the manufacturing of organoids with robotic automation, MIMETAS and Micronit created organoids on a chip (Thompson et al., 2020), and the American Type Culture Collection (ATCC) and Human Cancer Model Initiative (HCMI) intend to focus on cancer organoids for research and discovery. (Choudhury et al., 2020). These exciting collaborations between research and industry offer tremendous potential benefit for patients, yet the current governance framework in place limits benefit sharing with patients. The HIT cystic fibrosis (CF) Europe project is a key example. The trial screened the new drug Kalydeco on organoids derived from patients with CF that had novel mutations that the drug was not approved for, and a subset of these organoids responded well to treatment. (Boers et al., 2016; Choudhury et al., 2020; Hyun et al., 2020). However, the drug costs around US$275,000 per patient annually, and there was no plan to provide reimbursement to the original patients. (Boers et al., 2016). Lengthy negotiations were required that resulted in the obligation for adequate care to donors. (Boers et al., 2016). This highlights the importance of discussions surrounding reimbursement and critical evaluations of what obligations, ethically, researchers have in sharing benefits with the original donors of tissue. As organoid technology advances and private industry seeks to commercialize organoids for drug discovery and screening, we can only expect more situations like the CF trial. Researchers should consider ethical obligations to donors and draft a plan for benefit sharing prior to the consent process, so donors are aware of the infrastructure in place. (Boers and Bredenoord, 2018). Mostajo-Radji proposed the field of neurodiplomacy, which encourages multinational, interdisciplinary communication, education, and equitable voices in the development and access to technologic advances in the realm of neuroscience. This perspective will continue to increase in importance as organoid technology expands to ensure ethical scientific endeavors. (Mostajo-Radji, 2022).

## Conclusion

**FIGURE 2:**
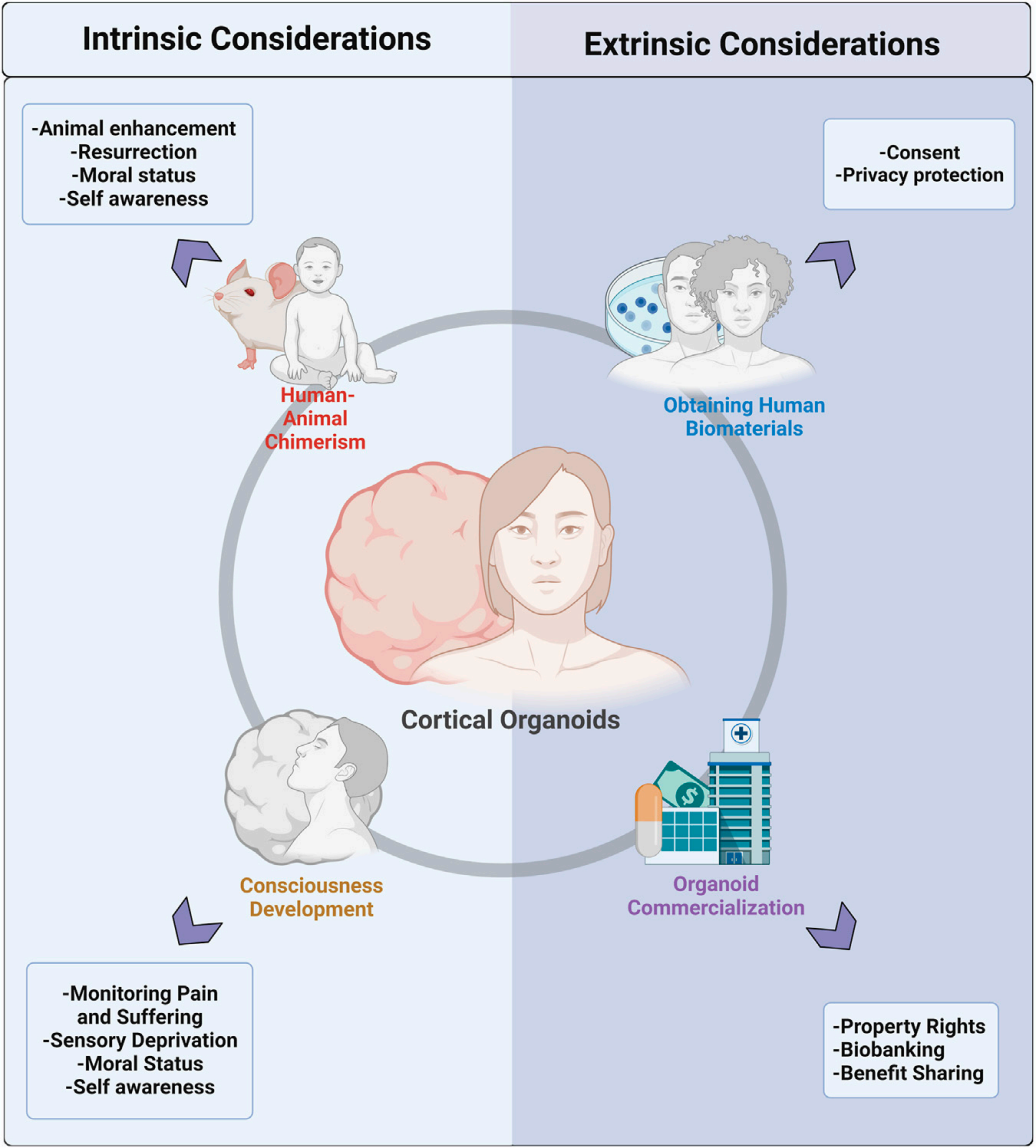
Pictural representation of the division of ethical and moral considerations for cerebral organoid technology into intrinsic and extrinsic factors. Intrinsic considerations include concerns that arise with the formation of human-animal chimeras and those that are associated with the potential for organoids to develop consciousness *in vitro*. Extrinsic considerations can be divided into concerns associated with the obtainment of human biomaterials needed to generate organoids and issues that arise with the growing commercialization of organoids.

In this review, we explore intrinsic and extrinsic ethical considerations in the realm of human brain organoid research (Figure 2), including ethical obtainment of human biomaterials, commercialization, chimera formation, and consciousness development. While it is possible that brain organoid implantation into NHPs can result in ‘enhanced’ animals and/or enhanced organoids, the ability of these chimeras to develop true self-awareness is questionable. Similarly, the ability of brain organoids *in vitro* to acquire consciousness is controversial; however, researchers are encouraged to study the biopsychological architecture of consciousness to monitor its potential development. Moving forward, it is critical to engage researchers, ethicists, and society in a conversation on what enhancements in chimeras and organoids are morally unacceptable. From this starting point, researchers can develop objective ways to test these enhancements. Moreover, with the rapid evolution of human tissue biotechnology and the entrance of organoids into the commercial market, we stress the adoption of ‘consent for governance,’ which shifts the ethical emphasis from a typical ‘consent or anonymization’ approach to a dedication to ongoing governance that emphasizes donor privacy and engagement, benefit-sharing, and ethical oversight. These changes are best adopted *via* close ongoing collaboration between researchers, bioethicists, donors, and companies. In the future, more research into donor preferences and values can guide the debate on fair governance infrastructure and elucidate donor interests in maintaining rights to their tissue in later applications. Organoid technology is an exciting step forward for uncovering mysteries of the human brain with great potential for clinical benefit. While it will likely be challenging to obtain consensus on the moral status of organoids and their downstream applications, we have an obligation to ensure the pursuit of scientific discovery is balanced with the careful consideration of ethics and morality.

## Data Availability

The original contributions presented in the study are included in the article/supplementary material, further inquiries can be directed to the corresponding author.
